# Democracy, Agony, and Rupture: A Critique of Climate Citizens’ Assemblies

**DOI:** 10.1007/s11615-023-00455-5

**Published:** 2023-03-07

**Authors:** Amanda Machin

**Affiliations:** Department of Sociology and Social Work, University of Agder (UiA), Universitetsveieen 25, 4630 Kristiansand, Norway

**Keywords:** Politics, Sustainability, Climate change, Disagreement, Agonism, Politik, Nachhaltigkeit, Klimawandel, Meinungsunterschiede, Agonie

## Abstract

Stymied by preoccupation with short-term interests of individualist consumers, democratic institutions seem unable to generate sustained political commitment for tackling climate change. The citizens’ assembly (CA) is promoted as an important tool in combatting this “democratic myopia.” The aim of a CA is to bring together a representative group of citizens and experts from diverse backgrounds to exchange their different insights and perspectives on a complex issue. By providing the opportunity for inclusive democratic deliberation, the CA is expected to educate citizens, stimulate awareness of complex issues, and produce enlightened and legitimate policy recommendations. However, critical voices warn about the simplified and celebratory commentary surrounding the CA. Informed by agonistic and radical democratic theory, this paper elaborates on a particular concern, which is the orientation toward consensus in the CA. The paper points to the importance of disagreement in the form of both agony (from inside) and rupture (from outside) that, it is argued, is crucial for a democratic, engaging, passionate, creative, and representative sustainability politics.

## Abstract (de)

Mattgesetzt von kurzsichtigen Konsuminteressen, erscheinen demokratische Institutionen nicht in der Lage, den Klimawandel nachhaltig zu bekämpfen. Immer häufiger werden deshalb Bürgerräte als ein Ausweg empfohlen, um dieser *Kurzsichtigkeit* der Demokratie zu begegnen. Ziel von Bürgerräten ist es, eine repräsentative Gruppe von Menschen mit unterschiedlichen Sichtweisen sowie Expert*innen aus unterschiedlichen Fachrichtungen zusammenzubringen, damit sie ihre Perspektiven auf ein komplexes Thema wie den Klimawandel miteinander austauschen. Indem sie die Gelegenheit für inklusive demokratische Deliberation bieten, versprechen Bürgerräte nicht nur, das Problembewusstsein zu schärfen, sondern auch, aufgeklärte und dadurch legitime Politikempfehlungen hervorzubringen. Allerdings mehren sich die Stimmen, die diese vereinfachenden und unkritisch positiven Einschätzungen in Frage stellen. Ausgehend von der agonistischen, radikaldemokratischen Demokratietheorie setzt sich diese Arbeit mit der Konsenserwartung auseinander, die an Bürgerräte gerichtet wird. Der Beitrag verweist auf die Bedeutung von Meinungsunterschieden und Konflikten in Form von Agonie (intern) und Disruptionen (von außen), die – so das Argument – für eine demokratische, engagierte, leidenschaftliche, kreative und repräsentative Nachhaltigkeitspolitik notwendig sind.

## Introduction

How can democracy and sustainability be reconciled? There is no straightforward answer to this question, and simply asking it immediately instigates a series of others. Such questions, however, should not be regarded as an impasse for political theory and democratic environmental policymaking, but can be seen more positively as an impetus for further debate. The introduction of this special issue calls for new questions in order to “stimulate a new research agenda” (Dietz, Fuchs, Schäfer, and Vetterlein 2023, in this issue). As they indicate, many respected scholars argue that the path to a more sustainable society is one that must be inclusive, participatory, and democratic (Barry 2002; Eckersley 2004, 2020; Fischer 2017). Against the claim that liberal democracy is simply a tool for the governance of “the condition of sustained unsustainability” (Blühdorn 2013, p. 18), political theorists as well as environmental activists suggest that frustration with existing political institutions does not have to lead to a complete rejection of democracy but could also be the basis for its radical transformation (Eckersley 2017).

Various proposals to enhance participation and to “green” democracy have been promoted (Machin 2022a). But one democratic innovation that has recently been given copious amounts of attention is the citizens’ assembly (CA). The CA brings together a representative group of citizens and experts from diverse backgrounds to exchange their different insights and perspectives on a complex issue such as climate change, and is therefore expected to facilitate a bottom-up movement of democracy (Dietz, Fuchs, Schäfer and Vetterlein 2023, in this issue). CAs on climate change are expected to inform citizens about this complex problem in order to help them imagine different ways of living and to formulate and legitimise robust environmental policies (Howarth et al. 2020, p. 1113). These forums have therefore been widely promoted by both environmentalists and political scientists (Dryzek et al. 2019; Howarth et al. 2020; Smith 2021). As one account states, “Citizen assemblies may not always generate the results that we expect, but by encouraging collective debate and decision-making they frequently propose a more positive future and different ways of getting there” (Howarth et al. 2020, p. 1113). Citizen assemblies on climate change have either taken place or are scheduled in numerous cities and countries,^1^ including Budapest^2^, Camden,^3^ Denmark,^4^ France,^5^ Geneva,^6^ Germany,^7^ Scotland,^8^ Spain,^9^ Krakow,^10^ Oxford,^11^ the United Kingdom,^12^ Vorarlberg,^13^ and Washington,^14^ and a “Global Citizens’ Assembly” took place in connection with the 2021 United Nations Climate Change Conference (COP26) in November 2021^15^ (see Table 1 for a comparison of some of these climate CAs).

**Table 1:** Comparison of four climate citizens’ assemblies (2020–2021)

	Participants	Meetings	Guiding question	Topics	Input	Outcome
**Climate** **Assembly UK**	108 assembly members, selected by sortition	Six weekends (three in Birmingham, three online) January–May 2020	*How should the UK meet its target of net zero greenhouse gas emissions by 2050?*	Underpinning principles for the path to net zero How we travel on land How we travel by air Heat and energy use in the home What we eat and how we use the land What we buy Greenhouse gas removals The changed context created by COVID-19 Additional recommendations	Presentations from 47 expert speakers as either informants or advocates	556-page report with key recommendations
**Scotland’s** **Climate** **Assembly**	105 assembly members, selected by sortition	Seven weekends of online sessions November 2020–March 2021	*How should Scotland change to tackle the climate emergency in an effective and fair way?*	Diet, land use, and lifestyle Homes and communities Travel and work	100 expert speakers selected by evidence group	204-page report with 81 recommendations
**Bürgerrat-Klima** **(Germany)**	160 assembly members	12 online sessions April–June 2021	*How can Germany achieve the goals of the Paris Climate Agreement with due consideration to social, economic, and environmental factors?*	Energy Mobility Buildings and heating Food and agriculture	Advised by experts from science, politics, and civil society	101-page report with official recommendations
**Washington Climate** **Assembly**	77 assembly members, selected by sortition	Twice-weekly online meetings, 5 h per week, over 7 weeks January–February 2021	*How can Washington State equitably design and implement climate mitigation strategies while strengthening communities disproportionately impacted by climate change across the state?*	Transportation Buildings Energy Natural solutions Circular economies Social policies Education and communication Governance	Presentations from experts and interested parties	123-page report with formal recommendations

However, as we will see, various critics offer important points regarding the limitations, even dangers, of any assumption that a deliberative forum such as the CA offers a straightforward solution to effectively and legitimately tackle an issue like climate change. These critics are wary of the suggestion that inequalities and hierarchies can be entirely removed from any political arena, and they accuse advocates of ignoring entrenched power relations as well as the role of passions and the question of how these forums connect with existing institutions.

This paper will elaborate on a particular concern, which is that climate CAs are oriented towards seeking consensus on a set of policy recommendations in a forum that is expected to be fully representative. This orientation, it is argued here, undermines the ineradicability and value of political disagreement. Drawing on agonist and radical democratic theory that sees disagreement as a constitutive feature of modern democratic societies, the paper will challenge the presupposition that political discussion and participation is obstructed by dissent and conflict. Disagreement in environmental politics allows alternative futures to be imagined, articulated, negotiated, and demanded, and it prevents the foreclosure of political questions around climate change (Pepermans and Maeseele 2018; Kenis and Lievens 2014; Machin 2020).

Disagreement, however, is manifested in different ways in democratic politics, and in this paper I therefore make an analytical distinction between two different—albeit connected—forms of disagreement. The first is *agony*, the type of contestation that potentially appears *inside* a political space or forum as the conflict between different demands, perspectives, and identities and that agonist theorists such as Chantal Mouffe and Bonnie Honig see as an ineradicable feature of politics. The second is *rupture*, which I understand as the manifestation of disagreement coming from *outside* in the form of a democratic excess, and is perhaps best described by Jacques Rancière. I target the displacement of disagreement in the *outcome* of the CA through the emphasis and celebration of the often fairly high levels of agreement reached in CAs, and also in the way this leads to its devaluation in the deliberative *process* itself, so that disagreement is rendered temporary and obstructive. The paper thus aims to contribute to theories regarding the nature and value of political disagreement, as well as to substantiate a critique of climate CAs.

I begin with a brief reminder of the ideals of deliberation followed by a description of the design of climate CAs. Next, I consider some of the various critiques of CAs before, focusing more specifically on the problematic preclusion of disagreement in climate CAs, in which, as I see it, the ultimate goal of consensus renders any form of dissent as temporary and obstructive. The paper concludes that disagreement in the form of both agony and rupture, which can contribute to a democratic, engaging, passionate, creative, and representative sustainability politics, is missing in climate CAs.

## Deliberation

It is not too much of an exaggeration to state that the majority of research that seeks to reconcile democratic participation with sustainability transformation has been informed by the deliberative school of democracy. This school, originating in the work of Jürgen Habermas and John Rawls, aims to base decisions on what Habermas refers to as “a public use of reason jointly exercised by autonomous citizens” (1994, p. 3). The claim here is that fair, inclusive, equal, and careful deliberation can generate informed and ethically and politically legitimate policy decisions and recommendations better than the strategising and bargaining of political elites. Deliberation requires citizens to go beyond their private self-interests and orient themselves to the public common good (Elster 1986; Rose 2009).

Some recommend that the interaction between political representatives is made more deliberative (Lidskog and Elander 2007, p. 88). More commonly, the emphasis is placed on a widespread change in political participation beyond voting in elections: “Ordinary people,” it is proposed, can be active participants in meaningful deliberation and are often able to overcome polarisation and heal deep divisions (Dryzek et al. 2019). Deliberative democrats therefore recommend the introduction of genuine opportunities for citizens from across society to engage in inclusive and thoughtful discussion. As John Dryzek and colleagues explain, deliberation involves “inclusive participation that encompasses citizens and leaders, mutual justification, listening, respect, reflection, and openness to persuasion” (Dryzek et al. 2019, p. 1145). Such collective deliberation is believed to be an effective mechanism for assuaging the “democratic myopia” that apparently renders democratic systems unable to tackle long-term and complex problems such as climate change (Willis et al. 2022). Numerous political thinkers and environmental activists therefore recommend deliberative democracy as the pathway towards sustainability (Baber and Barlett 2005; Barry 1999; Blue and Dale 2016; Christiano 2012; Dryzek 2000; Dryzek and Niemeyer 2019; Eckersley 2004; Hammond 2020a; Niemeyer 2013; Pimbert and Barry 2021; Smith 2003, 2021; Vlerick 2020; Willis et al. 2022), providing both ethical and epistemic justifications.

Deliberation is seen, first of all, as ethically valuable because it is supposed to encourage individuals to transcend their self-interests and become “other regarding.” David Miller refers to the “moralising effect of public discussion” (1992, p. 61), for narrow self-interest is difficult to defend in the public sphere. By individuals’ engaging in face-to-face discussions with their fellow citizens, deliberative democracy is expected to encourage a sense of community and awareness of others, including those to whom they initially regarded with suspicion or hostility (Vlerick 2020, p. 6). Deliberation encourages, too, an awareness of ecological concerns and dangers, broadens perspectives, and facilitates the public scrutinising of environmentally unsustainable practices. For Robyn Eckersley, although there is no guarantee that deliberation, or any other democratic procedure, will generate “green ends” (2017, p. 994), she nevertheless advocates deliberative democracy that, she states, “privileges generalizable interests over private, sectional, or vested interests, thereby making public interest environmental advocacy a virtue rather than a heroic aberration in a world of self-regarding rational actors” (2004, p. 117, see also Eckersley 2020, p. 220).

Second, and relatedly, deliberation is justified on the epistemic basis that it educates citizens and generates “better” decision-making (Estlund and Landemore 2018). As Simone Chambers writes, “[W]e humans … gain better cognitive results when thinking interactively and intersubjectively” (2018, p. 148). Participants learn about each other and themselves and therefore, the decisions they arrive at are supposed to be improved. “In the process of exchanging evidence related to proposed solutions, individuals discover information they did not previously have … deliberation is in itself a procedure for becoming informed” (Manin 1987, p. 349). Rebecca Willis and colleagues claim that deliberation emphasises “the power of the better argument” (2022, p. 5) and activates “system 2 thinking,” which is characterised by “more considered and reflective forms of judgement” (Willis et al. 2022, p. 4). Likewise, Dryzek and colleagues assert that “properly structured deliberation can promote recognition, understanding, and learning” (2019, p. 1145). Eckersley argues that deliberative democracy is particularly appropriate in the case of complex ecological problems; it makes it possible to expose the policies and interests of social, political, and economic elites to public scrutiny, and “a case can be made that deliberative democracy is especially suited to making collective decisions about long-range, generalizable interests, such as environmental protection and sustainable development” (Eckersley 2004, p. 118).

## Assembly

While some deliberative democrats demand a widespread change in public culture in general (Hammond 2020b), many promote the institution of various types of deliberative forums—known as deliberative mini-publics—in which citizens and various stakeholders, along with their representatives as well as scientists and different sorts of experts, are encouraged to exchange insights, perspectives, and knowledge in a process of mutual learning. As Graham Smith explains, “Deliberative mini-publics are participatory institutions in which randomly selected citizens learn, reflect, and deliberate on often complex and controversial areas of public policy before they come to make recommendations” (Smith 2021, p. 94). These forums include deliberative opinion polls, citizens’ juries, consensus conferences, planning cells, deliberative polling, and town meetings (see Gastil and Levine 2005; Smith and Setälä 2018; O’Flynn 2022).

These sorts of forums are expected to heighten public awareness of the long-term impacts of particular policies on the environment and to encourage innovative policy recommendations: “Such deliberative environments are seen as creative spaces in which new ideas and options can be fostered” (Hammond and Smith 2017, p. 15). Graham Smith warns that although these are not “a democratic panacea,” they are nevertheless “worthy of sustained consideration” (Smith 2021, p. 93). In his research, Simon Niemeyer, for example, has offered evidence that deliberation in a mini-public “improved the ability of citizens to better deal with the kind of complexity associated with climate change” (2013, p. 442).

One particular type of deliberative mini-public that has become increasingly prominent in discussions among both theorists and activists is the CA. Jonathan Rose provides a helpful definition: “A citizens’ assembly takes a group of random citizens of diverse ages, ethnic backgrounds, and socioeconomic status and after an intensive education programme followed by a public consultation and deliberation phase has them make a policy recommendation” (Rose 2009, p. 215). By bringing together a large group of ordinary people for lengthy periods, CAs are “extraordinary experiments” in deliberative democracy (Fournier et al. 2011, p. 13).

The design of the CA, according to Fournier and colleagues, echoes the councils of ancient Athens: “one of the most important institutions of Athenian direct democracy” (2011, p. 10). A CA typically involves 99–150 people and takes place over a series of weekends (Smith and Setälä 2018; Table 1). Participants are invited randomly by sortition (or “civic lottery”), and then great care is taken to sort from those who accepted the invitation a group that represents the wider population as closely as possible in terms of gender, age, ethnicity, class, region, and voting preference (Renwick 2017; Mellier and Wilson 2020). Participants might be given financial support to cover travel, accommodation, and childcare costs and access needs.^16^ They are then presented with input from experts from the fields of science, civil society, and industry, and with “carefully designed questions” (Capstick et al. 2020, p. 1). After lengthy deliberation, sometimes in smaller working groups, the assembly is normally expected to produce policy recommendations, which may then be handed to politicians or voted on in a referendum (Pal 2012). Some have advocated a CA at a global level (Dryzek, Bächtiger and Milewicz 2011), but most CAs take place at the state or local level.

CAs are recommended by political scientists who promote deliberative democracy as a mechanism that can produce a robust climate policy (Dryzek et al. 2019). Howarth and colleagues agree that “deliberative engagement mechanisms, such as citizens’ assemblies and juries, could be a powerful way to build a social mandate for climate action post-COVID” (2020). Recently, activists too, most prominently the protest movement Extinction Rebellion, have expressed a strong demand for “the government to create and be led by the decisions of a citizens’ assembly on climate and ecological justice” (Extinction Rebellion 2019, p. 5), which they say will “provide an opportunity to explore the views of a broadly representative sample of people in fair and equitable way” (2019, p. 7).^17^ Indeed, as the growing list of climate CAs indicates, there is a definite trend towards the use of CAs by national and local governments.^18^

Empirical research has been undertaken on CAs in Canada (Warren and Pearse 2008; Pal 2012; Fournier et al. 2011), Ireland (Carolan 2018), Australia (Carson 2007), France (Giraudet et al. 2021), the UK (Flinders et al. 2016; Wells et al. 2021), and the Netherlands (Boogard and Binnema 2017). This body of research has produced mixed conclusions. The CAs are widely reported to be “a positive experience” for those involved (Cain and Moore 2019, p. 13), and some suggest that although CAs are “a significant investment in terms of money, time, energy and relationship building,” they also should be seen as “a positive social investment that is likely to increase the efficiency of subsequent policies and decisions” and that can drive a “broader public debate about an issue, challenge or event” (Flinders et al. 2016, p. 3). Others claim that they often fail to enhance “a wider public conversation” and that their impact on policymaking is difficult to assess (Wells et al. 2021, p. 5).

Certainly, critical voices warn about the simplified and celebratory commentary surrounding the CA (Carolan 2018; Lafont 2015). This type of forum invites numerous questions not only concerning its most effective design and practical implementation and the way it fits within the political apparatus of liberal democracies, but also with regard to how such an institution may reinforce specific understandings and processes of democratic participation and undermine others and how this overlooks, if not reinforces, the authority of certain types of bodies and particular forms of knowledge. It is to these critiques that I turn to next. In the following sections, I draw on academic work and also reports of the CAs themselves in order to illustrate and test critical arguments.

## Critique

Critics of CAs raise concerns about the quality of political participation that can occur within deliberative mini-publics. Practical problems of selecting participants and information aside, there remains the issue of power relations in these forums, which provokes Albena Azmanova to ask, “How do we know that public deliberations are really free of power asymmetries, ideological idiom, and various forms of manipulation?” (2010, p. 48). The process is supposed to be neutral, but it normally starts with a “learning phase” (Willis et al. 2022) in which invited experts are expected to give presentations that deliver what is described as “foundational knowledge” (Bürgerrat-Klima 2021, p. 12; Scotland’s Climate Assembly 2021, p. 6). It is at least possible that participants find it difficult to challenge knowledge claims that are said to be foundational. Lynn Sanders believes that the deliberative model of democracy “does not take sufficient account of the ways that status and hierarchy shape patterns of talking and listening to ensure that all perspectives are considered” (Sanders 1997, p. 370). Certain types of speaking are given priority in deliberative forums, which might mean that certain perspectives, communicated in different ways, are not heard (Young 1996). There is, therefore, a concern that some bodies dominate in a deliberative space because habituated expectations and perceptions allocate the authority of particular bodies over others (Machin 2022b, p. 75). This might not only skew the discussion but also restrict the possibility for the expression of a diversity of opinions and lived experiences and the emergence of new ideas.

Some proponents of deliberative democracy are themselves not convinced by the growing emphasis on mini-publics. They are concerned that these circumscribed “experimental” spaces of deliberation that are controlled by governments do not constitute an instrument for citizens to take the initiative themselves to challenge the status quo; the topic for discussion, the timing, the material, and other practical details are entirely arranged “top down” by the authorities. This can force participants into what deliberative democrat proponent Marit Hammond (nee Böker) calls “an ominously passive role” (Böker 2017, p. 11). Indeed, observers have noticed that CAs “do not deliver breakthrough ideas, but are rather in line with previously adapted policies” (Ufel 2021, p. 88). Hammond emphasises the importance of a broad deliberative culture and the promotion of “socio-political spaces of inclusive critical engagement” (Hammond 2020a, p. 188; Hammond and Smith 2017, p. 23), and it is not clear that this is encouraged by CAs. Likewise, Chambers states that the “protected sphere” of the forum can only be one part of the picture of the broader democratic public arena (Chambers 2018). Others highlight the fact that politicians not only influence the process but are able to cherry-pick and simply ignore the recommendations if they do not fit with their agenda (Ufel 2021, p. 86).

Another critique arises from the putative aim of the CA to create a space for sober rational reflection and exchange. Deliberative democrats suggest that while emotions “play an important role in making reasons vivid,” they can “distort our capacity for careful reasoning and reflection” (O’Flynn 2022, p. 54). It can be asked, however, whether an emphasis on reason over emotion might undermine the passionate exchange of ideas that engages participants in politics (Walzer 2004). Cheryl Hall makes the important point that although a “supreme value” may be placed on “calm rational discussion” in deliberative forums (2007, p. 81), this actually belies the passions that underpin deliberation: “[D]eliberation already involves passions” (Hall 2007, p. 92). As she notes, the privileging of rational discussion “maintains the power of those who are already dominant because they are the ones who have perfected the art of appearing rational” (Hall 2007, p. 83). Emotions cannot be easily bracketed off political exchange, and “the issues that most people want to talk about are not always easy to articulate in the format of rational reasoned argument” (Hayward 2020, p. 123). While emotions do clearly pervade and inform the interactions between assembly members—in their comments, participants refer to emotions of pride as well as disappointment and to their “impassioned,” “compassionate,” “intensive,” and “inspiring” discussions^19^—the importance of this aspect of political interaction does not seem to be acknowledged in the deliberative process of the climate CAs, which is described as careful and considered (Scotland’s Climate Assembly 2021, p. 102), and which requires the provision of “a sufficient amount of time for reflection … necessary to achieve well-thought-out decisions” (Washington Climate Assembly 2022, p. 4).

But if politics is emotional and passionate, it also unpredictable and unruly. Political interaction is as prone to tensions and frictions as it is to harmony and accord. This is not to say that disagreement is irrational or that rationality has to be dispassionate, but simply that the portrayal of arriving at a rational agreement through deliberation offers not only a very insipid picture of politics but also a very distorted one. Conflicts reveal and heighten the nonrational aspects of political life, and this does not make them illegitimate or dissolvable but, on the contrary, shows their durability and their capacity to enliven and engage. To try to overcome disagreement is to misconceive democratic politics, or, in the words of Bonnie Honig, it is to ultimately *displace* politics by assuming that success lies in the eradication of conflict and struggle (Honig 1993, p. 2). In the following section, I offer a critique of CAs that challenges their orientation towards the goal of consensus and the way that this dampens the possibility of democratic disagreement in both the outcome and the process of deliberation. It is disagreement, I argue, that facilitates a lively, passionate, creative, engaging, and representative sustainability politics. As Aletta Norval notes, disagreement takes different forms (2007, p. 39). Democratic disagreement can take the form of *agony* that enlivens from the *inside*, and it can take the form of *rupture*, which disrupts from the *outside*. As I show in the next sections, climate CAs not only fail to harness these forms of disagreement but tend to actively work against them.

## Agony

Often contrasted to the deliberative school of democracy is the agonistic school, characterised by its emphasis on the plurality, uncertainty, and tragedy of politics that is constituted by perpetual struggle. Agonists suggest that political disagreement is not only inevitable but can enliven and enrich democratic politics (Connolly 2005, 2013; Honig 1993; 2009; Mouffe 1999, 2005; Tambakaki 2017). They are, therefore, wary of assertions of the possibility of reaching a full consensus through deliberation—however inclusive, equal, and fair it is intended to be. Agonism, explains Paulina Tambakaki, stresses the central role played by contestation between differences, which sustains openness, pluralises politics, and, as she puts it, *renews* democracy (2017, p. 579). Agonists do not call for any radical overthrow of liberal democratic institutions, but they do insist upon the opening up of those institutions to differences that have hitherto been suppressed (Tambakaki 2017, p. 579). Disagreements should not, therefore, be understood as irrational blockages or lamentable failures, as they are commonly presented in accounts of deliberative democracy and CAs, but rather as legitimate expressions of difference that both expose closure and exclusion and open up the possibility of alternatives. An assertion of consensus can provide a useful cover for powerful stakeholders who may be loathe to see a change to the status quo (Ward et al. 2003, p. 287). Scholars of environmental politics, drawing on the agonistic school of thought, have thus argued against what they see as depoliticisation and the encroachment of the postpolitical discourse on climate politics. The invocation of the need for consensus, as Anneleen Kenis and Matthias Lievens note, is a political manoeuvre that tries to disguise the politics around climate change (2014). Repoliticisation, on the other hand, “brings different voices to the fore” and therefore “helps to open the door for real and effective change” (Kenis and Lievens 2014, p. 545).

Climate change appears to be an issue that is particularly liable to disagreement between positions that are situated in different lived existences (Machin 2013, 2015, 2020). But if consensus is unlikely, it is also not necessarily salutary. It is possible that democratic debates and forums can be enriched and enlivened through the clash of perspectives over environmental issues, and further that environmental policymaking might ultimately be better served through the recognition and appreciation of political disagreement. This “ecological agonism” approach suggests that the disagreement provoked by environmental hazards can both engage citizens in political debate and facilitate the emergence and consolidation of socioecological alternatives. Conventionally marginalised perspectives may contain valid and valuable insights and ideas (Machin 2020). After all, as Chantal Mouffe points out, it is only through political discord that prevailing power structures can be reconfigured (Mouffe 2005, p. 21).

Arguably, then, it is the value placed on political contestation that marks the line between the deliberative and the agonistic schools. Early theories of deliberation certainly eschewed conflict and emphasised the importance of its participants’ reaching consensus. Indeed, for Habermas, it was the “orientation to the goal of communicatively reached agreement” that brought participants together to deliberate in the first people (Habermas 2002, p. 44; see also Estlund and Landemore 2018). Later (or “second-generation”) theorists have recognised the problem with this ideal and have moved towards a recognition and even an appreciation of the role of difference and disagreement and the “unfinished and open-ended aspect that serves as the lifeblood of democracy” (Ercan and Gagnon, 2014, p. 7; Ufel 2021, p. 80).

And yet this respect for differences and disagreements is not so evident in the guides, descriptions, and evaluations of CAs. Consensus is generally seen in this literature as a normative *goal* of deliberation as well as a practical *outcome* of the assemblies, and is the desirable stopping point beyond which discussion does not have to be pursued.^20^ The emphasis on consensus is remarkable in explicit statements such as “the premise of the CA is to look for a consensus” (Gerwin 2018, p. 80), “the importance of talk that is framed around consensus” (Rose 2009, p. 230), and “supportive structures to aid consensus-building” (Cain and Moore 2019, p. 36). Decisions in CAs are expected to be “consensus based” (Rose 2009, p. 216), and CAs are supposed to help with “forging consensus” (Climate Assembly UK 2022, p. 4) and allowing governments to forge a “cross-party consensus” (Climate Assembly UK 2022, p. 7). The Global Citizens’ Assembly expressed confidence that it can “build consensus” (Global Assembly Team 2022, p. 2), and the report on the Scottish Climate CA highlighted the “overwhelming consensus” of participants who supported the final set of recommendations (2021, p. 7). Analysts reporting on the climate CA in Camden, London, noticed the changes in the participants’ confidence, behaviour, and identification, but wrote that “this is not the point of a CA—they are deliberative processes aiming to build consensus and legitimacy around responses to contested policy issues” (Cain and Moore 2019, p. 36). A group of scholars analysing the French Convention noticed the orientation towards consensus: “Reaching a consensus, as measured by the absence of explicit dissent, was systematically favored over voting by the organizers” (Giraudet et al. 2021, p. 12).

Indeed, throughout the literature on climate CAs, despite the occasional acknowledgement of “nuanced discussions” (Climate Assembly UK 2022, p. 5) and of “making room for disagreements and differences of opinion” (Scotland’s Climate Assembly 2021, p. 102) and the observation that “disagreements were not uncommon” (Bürgerrat-Klima 2021, p. 82), there is a greater emphasis placed on “consensus building” (Scotland’s Climate Assembly 2021, p. 136) and on the achievement of agreement on the recommendations, which are almost always regarded and celebrated as the definite main outcome of the CA. These recommendations come “towards the end of the process” and are “jointly formulated” (Bürgerrat-Klima 2021, p. 76). The purported goal of the CA in general, then, is definitely not *to find, highlight, and understand the main points of contention* over climate change, but rather *to seek the principles and recommendations on which the assembly participants could agree*. For example, the Climate Assembly UK stated that “the assembly members’ agreement of their additional recommendations brought the assembly process to its close” (Climate Assembly UK report 2022, p. 551), and the Washington Climate Assembly report explains that its goal was to provide “a set of broadly supported climate mitigation recommendations” for the state legislature (Washington Climate Assembly 2022, p. 8). Perhaps even more revealingly, the Oxford CA on Climate Change report states, “There was widespread belief that Oxford should be a leader in tackling the climate crisis … However, it’s important to consider the caveats to this broadly optimistic and positive image … there was little consensus on when before 2050 ‘net zero’ should be achieved” (Ipsos MORI 2019, p. 3). Consensus is regarded as an unquestioned good and a finality, whereas disagreement is seen as temporary, obstructive, and unfortunate.

To be clear here, my point is not that these reports falsely claim that full consensus exists (which would simply not be true) but rather that many of them simply make the assumption that the closer to consensus the assembly gets (or, to be accurate, the higher the percentage of members who come to an agreement), the better. The reasons for dissenting are not analysed or differentiated but are lumped together and relegated to the part of the percentage that is *negatively* expressed. Consider that the reports from Climate CAs in Germany, Washington, Scotland, Camden, and the UK append to their list of recommendations the number or percentage of the Assembly who had voted for each of them, signalling the preoccupation with the *quantity* of agreement on a recommendation, which is presumably meant as an indication of its validity and legitimacy. This quantity rarely, if ever, reaches 100%—it is actually asserted that 80% qualifies as “almost a complete consensus” (Gerwin 2018, p. 22), which suggests that that differences *do* persist but are rendered virtually invisible in the 20% that goes unanalysed.

Consensus, moreover, is seen as a result of the participants becoming more informed by listening to expert presentations and reflecting amongst themselves. As Giraudet et al. note, in the French Citizens’ Convention for Climate, “the generally high rate with which measures were approved was sometimes celebrated as evidence that giving citizens the appropriate scientific background was sufficient to generate informed and consensual decisions” (2021, p. 13). They make the important point that the invited experts each gave their presentations in turn and were not given the opportunity to question each other’s evidence and claims; potential disagreements between the different experts themselves were thereby structurally precluded (Giraudet et al. 2021, p. 12).

To say that greater value is placed on agreement than on disagreement here is to understate the way in which CAs seek consensus and to displace the possibility of valid and ongoing disagreement between the assembly members. This goal of consensus has the effect of underestimating and stifling the tenacity and value of disagreement in the discussions. There is no space inside the CA for the political *agon*, in which opponents vie to articulate and consolidate distinctive positions that might offer an array of valid alternative strategies and imaginaries for socioecological transformation. There is no room for presenting clearly delineated and distinct options. There is no formal place for opposition. Instead, the participants are expected to be united around a common concern in a shared world. “The deliberative nature of the CA,” write Geerten Boogard and Harmen Binnema, “… stimulates citizens to look for what they share (common ground) instead of what separates them” (Boogard and Binnema 2017). In a video about the Global Assembly, the narrator refers to hearing the “voice of humanity”^21^—as if this would consist of one single and unified sound. Azmanova expresses suspicion about the claims of deliberation to represent the “public voice” and suggests instead that a forum of public deliberation is more convincingly understood to be “a place where social conflict is communicatively enacted” (2010, p. 52). In climate CAs, however, conflicts are generally not made explicit but are, on the contrary, rendered invisible behind the pursuit of a list of recommendations; the aim is not to bring to light “the multiple social conflicts that make up the fabric of the societies we inhabit” (Azmanova 2010, p. 42) but rather to increase and report the quantification of agreement.

As we can see, then, in the climate CA a group of representative participants are expected to overcome the agony of differences and to unite around a common concern in a shared world. But the preselection both of the participants and of the “common concern” points to the foreclosure of another form of disagreement, rupture, that I consider in the next section.

## Rupture

A great deal of care is taken with the sortition process of climate CAs to ensure that participants are as descriptively representative of society as possible (Willis et al. 2022, p. 6). After invitations have been sent randomly to households, participants are then selected, from those who accept the invitation, on the basis of criteria—such as age, gender, ethnicity, disability, household income, educational level, migration experience, geography, rurality, and attitude to climate change—in order to reflect the demographic composition of the wider population as a whole. In this way, by involving people from “all walks of life,” the process creates “a city or country in miniature” (Gerwin 2018, p. 17).^22^ Such representativeness is expected to improve the quality of the deliberation inside the CA and to create legitimacy for its process and outcomes. It is also supposed to make CAs more representative of “the people” than existing governments are. This is precisely why Extinction Rebellion demand the creation of a CA at the national level: “Deliberative processes, supported by safeguards against bias, lead to more diverse and informed voices in political debates than in a purely elected body, such as the House of Commons” (Extinction Rebellion 2019, p. 7).

The assumption that a climate CA is fully representative, however, can be challenged on various grounds. The fact that it is to some extent *descriptively* representative (it “mirrors” the population in its composition of individuals in terms of features such as gender, ethnicity, and age) does not mean it is necessarily *substantively* representative (reflecting the various substantive interests of the population). Organisers of a CA cannot know for sure whether all political positions on climate have been included. Moreover, the decision about which particular descriptive features are to be mirrored is, to some extent, a contingent one. It is not hard to think of characteristics that are not deemed relevant to be represented (body size, time spent in prison, IQ, mental health, and so on). And one can list easily enough those individuals whose opinions and experiences might be highly pertinent but whose voices are not included (children, future generations, distant others, nonhuman nature).

This is not solely a practical matter of opening up the forum to those whose exclusion is recognised, but of acknowledging the exclusions that go unrecognised *precisely because* of the claim that everyone is included. For Rancière, exclusion does not consist of being left out; it pertains to “the very invisibility of the partition” (1999, p. 116). The claim of full representativeness excludes the possibility of exclusion and stifles any possibility of new claims of representation forming outside the forum that delineate distinct conceptions of political boundaries and subjects. But it is actually the disruptive formation of new political subjects that, for Rancière, constitutes democracy (1999, p. 101, 2004, p. 5). Politics really begins for Rancière not when the already counted confront each other but when those with “no part” interrupt the dominant order by “part-taking” (1999, p. 18). He writes, “[P]olitical subjects are, thus, not representatives of parts of the population but processes of subjectivation which introduce a disagreement, a dissensus” (2004, p. 6). Disagreement here is not a dispute between already existing parts but rather the disruption of the established political order that “undoes the given” (Tambakaki 2009, p. 104). It is this disagreement that I call rupture.

Rupture constitutes a form of disagreement that is distinct, I suggest, from the agonistic contest or struggle *between* differences *inside* the political assembly. Theories of agonism—which tend to focus on renewal of the institutions of liberal democracy rather than their radical transformation—can be criticised for underestimating the extent of contestation needed to instigate and sustain substantive change (Tambakaki 2017, p. 587). Rupture, in contrast, disturbs the claims of representation and corresponding social imaginaries that form the foundations of the political assembly from the outside. Consensus here is rejected, but not in the form of the goal, outcome, or endpoint of the CA; what is rejected here is the suggestion of consensus as the *starting point *of the CA. “What indeed is consensus,” asks Rancière, “if not the presupposition of inclusion of all parties and their problems that prohibits the political subjectification of a part of those who have no part, of a count of the uncounted?” (1999, p. 116).

Climate CAs are supposed to *themselves* actually constitute the *demos* who can part-take to rupture the unsustainable status quo. Willis and colleagues point out that the participants in the CA might “ask the difficult questions” eschewed in formal policymaking (2022, p. 9). For Extinction Rebellion, the exclusion of many people from politics is the very problem they seek to rectify with CAs (Extinction Rebellion 2019, p. 8). This is a hopeful and inspiring claim, but can climate CAs really challenge the very regime by which they have been instituted? Can they dissolve the claim of their own representativeness and install a new political subject who is capable of undermining the dominant framing of the problem of climate change, who can rebuild the stage on which it appears, and who can destabilise the prevailing common sense that is often steeped in neoliberal capitalist relations and subsists with human–nature dualism? Is it not significant that the starting questions for climate CA tend to be questions that ask *how*, and not *if*, a policy strategy or target should be reached, and therefore do not allow for the fundamental challenging of that strategy or target (see the guiding questions in Table 1)?^23^ What is at stake here is the possibility of disagreement coming from *outside* the CA—political dissent that might rupture the presuppositions that form the background and the starting questions to deliberations but that often go unnoticed.

To be clear, I am not claiming that the participants in the CA do not come from a variety of different socioeconomic backgrounds (which they clearly do).^24^ Nor am I suggesting that the disruption of a political space always results in its democratic and progressive transformation (which it clearly does not). My concern, rather, is that to the extent that a CA closes off a political space based on the claims that it is fully representative and disavows the possibility of rupture, it forecloses an important dimension of democracy. In general, the climate CA is entirely closed; its agenda and process are predetermined, and rupture is disallowed and delegitimised.^25^ Thus, instead of facilitating democratic politics over climate change, CAs can actually end up working against it.

## Conclusion

In the context of a changing climate that both stems from and exacerbates socioeconomic inequalities and injustices, it seems crucial to attend to the way that institutions, ideals, and practices of democracy might be reimagined and rebuilt more sustainably (Fischer 2017, p. 3; Machin 2022a). The CA is widely promoted as a new democratic instrument for both enhancing democratic participation and reconciling democracy with sustainability. It is expected to increase public awareness about environmental concerns as well as to legitimise robust environmental policy. At the very least, these forums “challenge the myth that people are irredeemably disengaged from politics” (Flinders et al. 2016, p. 2).

I have argued, however, that there are dangers in regarding the climate CA as a mechanism for a democratic climate politics. The assumption of full representativeness makes it difficult to rupture the starting questions, assumptions, and identities of the CA, and its emphasis on reaching consensus dampens the agony of difference. If the unsustainable status quo is to be challenged, then environmental politics needs to offer and support new and distinctive ideas. And it is precisely through rupture and agony, I suggest, that alternative pathways, perspectives, and coalitions on climate change can circulate, align, and consolidate. The disagreement that arises from the expressions of difference that emerge from both *inside* and *outside* political forums should be celebrated. But the orientation of the climate CA towards consensus and the quantification of the agreement as a signal of approval works to construct disagreement in the process of deliberation as a temporary and unfortunate hindrance. In their present form, by undermining the potential for the emergence of alternatives, CAs might actually *obstruct* sustainability transformation.

What might be the alternative to a CA that is oriented towards consensus? One response might be that CAs can be designed to draw attention to political conflicts between socioecological alternatives and to detect and communicate, rather than ignore, the main points of disagreement over climate change. Rather than seeking and measuring agreement on environmental policies, emphasis would be placed on locating, highlighting, and grasping the issues on which citizens disagree. A more radical response is to give up on the construction of artificial deliberative forums and to instead champion existing social and political movements that themselves offer alternative imaginaries of democracy and sustainability (Machin 2022c).

Certainly, if there is to be a genuine sustainability politics, then democrats should refrain from demanding consensus, quantifying agreement, and reducing political participation to deliberation. Instead, they should call for an agonistic and disruptive politics in which participants are passionate, the remainder are remembered, and the assembly is animated by disagreement.

## Footnotes

1 For a more extensive (but probably not exhaustive) list of citizens’ assemblies on climate change as well as other topics, see https://www.buergerrat.de/en/background/citizens-assemblies-worldwide/

2 https://demnet.hu/en/citizens-assembly-in-budapest-2020/

3 https://www.camden.gov.uk/citizens-assembly-climate-crisis

4 https://tekno.dk/project/citizen-assembly-at/?lang=en

5 https://www.conventioncitoyennepourleclimat.fr/en/

6 https://oidp.net/en/practice.php?id=1285

7 https://buergerrat-klima.de/english-information

8 https://www.climateassembly.scot/impact

9 https://asambleaciudadanadelcambioclimatico.es/

10 https://ipp.expert/krakowski-panel-klimatyczny/

11 https://www.oxford.gov.uk/info/20011/environment/1343/oxford_citizens_assembly_on_climate_change

12 https://www.climateassembly.uk/

13 https://vorarlberg.at/-/bürgerrat-klima-zukunft

14 https://www.waclimateassembly.org/

15 https://globalassembly.org/declaration

16 For example, the UK and Washington climate CAs gave financial compensation in the form of honoraria or stipends

17 https://xrcitizensassembly.uk/

18 https://europeanclimate.org/stories/the-growing-traction-of-climate-citizens-assemblies/

19 These emotions appear in the comments of the participants who are reflecting on their participation, included, for example, in the reports of the German and Scottish climate CAs (Bürgerrat-Klima 2021; Scotland’s Climate Assembly 2021) and the analysis of the Camden climate CA (Cain and Moore 2019).

20 Estlund and Landemore (2018) note that in deliberative theories, consensus might be viewed as a goal, an outcome, or a stopping point. My analysis indicates that this applies in the case of climate CAs.

21 https://globalassembly.org/

22 The CA is described as “a microcosm of the state” (Washington Climate Assembly 2022, p. 3) or a “mini-Scotland” (Scotland’s Climate Assembly 2021, p. 102).

23 Thanks to Alexander Ruser for this point.

24 But note that the report of the climate CA in Germany admits that people with low educational attainment and people who did not report the issue of climate protection as important to them were underrepresented (Bürgerrat-Klima 2021, p. 82).

25 The Washington climate CA, held online, encouraged the public to observe and make suggestions (Washington Climate Assembly 2022, p. 20) but although this might make the assembly more “open,” it still does not allow for the “rupture” of its background rationale and starting point.
